# Autophagy is required for proper meiosis of porcine oocytes maturing *in vitro*

**DOI:** 10.1038/s41598-018-29872-y

**Published:** 2018-08-22

**Authors:** Xing-Hui Shen, Yong-Xun Jin, Shuang Liang, Jeong-Woo Kwon, Ji-Wei Zhu, Lei Lei, Nam-Hyung Kim

**Affiliations:** 1 Department of Histology and Embryology, Harbin Medical University, Harbin, Heilongjiang China; 2 Department of Animal Sciences, Chungbuk National University, Cheongju, Chungbuk Republic of Korea; 3 Department of Animal Science, College of Animal Sciences, Jilin University, Changchun, Jilin, China; 4 Department of Forensic Medicine, Harbin Medical University, Harbin, Heilongjiang China

## Abstract

Autophagy is an essential cellular mechanism that degrades cytoplasmic proteins and organelles to recycle their components; however, the contribution of autophagy during meiosis has not been studied in porcine oocytes maturing *in vitro*. In this study, we observed that the autophagy-related gene, *LC3*, was expressed in porcine oocytes during maturation for 44 h *in vitro*. Knockdown of the autophagy-related gene, *BECN1*, reduced both BECN1 and LC3 protein expression levels. Moreover, BECN1 knockdown and treatment with the autophagy inhibitor, LY294002, during maturation of porcine oocytes *in vitro* impaired polar body extrusion, disturbed mitochondrial function, triggered the DNA damage response, and induced early apoptosis in porcine oocytes. Autophagy inhibition during oocyte maturation also impaired the further developmental potential of porcine oocytes. These results indicate that autophagy is required for the *in vitro* maturation of porcine oocytes.

## Introduction

*In vitro* production of porcine embryos is a valuable method used for biomedical research and agricultural applications. As the physiology, anatomy, pathology, genome organization, body weight, and life span of pigs are similar to those of humans, the domesticated pig represents an alternative biomedical model to rodents for research into specific human diseases^1,2^. The viability of porcine embryos produced *in vitro* is determined by the quality of *in vitro* matured oocytes, which directly influences subsequent embryo development^3^. Therefore, it will be advantageous to improve the quality of porcine oocytes by identifying the developmental events that occur during their *in vitro* maturation (IVM).

Autophagy is a basic process involved in degrading unnecessary or dysfunctional cell components^4,5^, and has a key role in various physiological processes, including adaptation to starvation, quality control of cytoplasmic constituents, and clearance of intracellular pathogens^6,7^. The autophagy process begins with the engulfment of targeted components, including macromolecules and organelles (e.g., mitochondria, peroxisomes, and endoplasmic reticulum) in double-membrane bound autophagosomes^4,5^. Autophagy reduces cell stress by eliminating damaged mitochondria, controlling reactive oxygen species production, and reducing apoptosis^8,9^. Damaged mitochondria may produce elevated levels of reactive oxygen species (ROS) leading to DNA damage, which can occur either before or after nuclear envelope breakdown during meiosis^10^. During autophagy, a cytosolic form of microtubule-associated protein 1 light chain 3 (LC3) (LC3-I) is conjugated to phosphatidylethanolamine to form LC3-phosphatidylethanolamine conjugate (LC3-II), which is recruited to autophagosomal membranes^11^. As the amount of LC3-II correlates well with the number of autophagosomes^12^, its levels are widely used to monitor autophagic activity. In addition, beclin 1 (*BECN1*), the mammalian ortholog of yeast *Atg6*, was the first mammalian gene identified as having a role in autophagy^13,14^.

The earliest autophagic event observed in mammalian development is in fertilized oocytes^15,16^. In mouse, ATG5-deficient oocytes fail to develop beyond the four-to-eight-cell stage after fertilization with ATG5-deficient sperm. Moreover, in pigs, treatment of preimplantation stage embryos with 3-MA (an autophagy inhibitor) or rapamycin (an autophagy activator) influences maternal mRNA degradation and induces apoptosis. Hence, autophagy is essential for the preimplantation development of mouse and porcine embryos. In contrast, the role of autophagy during oocyte maturation is largely unknown. LC3 is not detected in unfertilized mouse and rat oocytes *in vivo*^15,17^. In contrast with findings in mice and rats, LC3-II is present in porcine oocytes cultured *in vitro*^18^; however, autophagy function prior to fertilization has not been studied in porcine oocytes matured using *in vitro* systems.

In this study, we investigated the effect of autophagy in porcine oocytes during *in vitro* maturation. Using both a specific inhibitor (LY294002) and an RNA interference strategy, we examined the role of autophagy during porcine oocyte maturation *in vitro*, through analysis of mitochondrial membrane potential, DNA damage, apoptosis, and subsequent embryonic developmental. Our results provide evidence that autophagy affects oocyte quality in this *in vitro* porcine system.

## Materials and Methods

All chemicals were purchased from Sigma-Aldrich Co., Inc. (St. Louis, MO, USA) unless otherwise indicated. All manipulations were performed on a heated stage adjusted to 38.5 °C unless otherwise indicated.

### Oocyte Collection and Culture

Porcine ovaries were provided by a local slaughterhouse (Farm Story Dodarm B&F; Umsung, Chungbuk, Korea) and were transported to our laboratory at 25 °C in Dulbecco’s PBS, supplemented with 75 μg/L penicillin G and 50 μg/L streptomycin sulfate. Cumulus-oocyte complexes (COCs) were aspirated from follicles (approximately 2–8 mm in diameter) and washed three times with 4-(2-hydroxyethyl)-1-piperazineethanesulfonic acid (HEPES)-buffered Tyrodes medium containing 0.1% (w/v) polyvinyl alcohol (PVA). Collected COCs were matured in tissue culture medium 199 (TCM199) (Gibco) supplemented with 0.1 g/L sodium pyruvate, 0.6 mM L-cysteine, 10 ng/ml epidermal growth factor, 10% porcine follicular fluid (v/v), 10 IU/ml luteinizing hormone, and 10 IU/ml follicle-stimulating hormone for 44 h at 38.5 °C in 5% CO_2_ and humidified air. After maturation, cumulus cells were removed by pipetting in the presence of 0.1% hyaluronidase (w/v) for 2–3 min.

### Oocyte Activation and Embryo Culture

Oocytes were activated parthenogenetically using an electrical pulse (1.0 kV/cm for 60 msec) in activation medium (280 mM mannitol, 0.01 mM CaCl_2_, and 0.05 mM MgCl_2_), followed by 3 h of incubation in PZM-5 medium containing 2 mM cytochalasin B (Sigma). Embryos were then washed several times in PZM-5 supplemented with 0.4% (w/v) bovine serum albumin (BSA) and cultured in a humidified atmosphere of 5% CO_2_ and 95% air at 38.5 °C.

### Preparation of Double-Stranded RNA Targeting *BECN1*

For knock-down of *BECN1* expression, we designed a double-stranded RNA (dsRNA) targeting its mRNA sequence. *BECN1* DNA amplification was performed using cDNA synthesized from RNA isolated from 30 porcine blastocysts by 1st stranded synthesis (Legene, San Diego, CA, USA) and a Dynabeads mRNA direct kit (Life technologies AS, Oslo, Norway). *BECN1* dsRNA primer sequences were, 5′-TAATACGACTCACTATAGGGAGACCACAACCTCAGCCGAAGACTGAA-3′ and 5′-TAATACGACTCACTATAGGGAGACCACTTTCAGGCCCATCTTATTGG-3′, including the T7 promoter sequence. The amplified *BECN1* DNA fragment was purified by electrophoresis and gel extraction and *in vitro* transcription of *BECN1* dsRNA was performed using the MEGAshortscript T7 kit (AM1354, Ambion, Austin, TX, USA). *BECN1* dsRNA transcripts were purified using phenol-chloroform extraction and isopropyl alcohol precipitation, and stored at −70 °C until microinjection.

### Microinjection

Microinjections were completed within 1 h using an Eppendorf microinjector and a Nikon Diaphot ECLIPSE TE300 inverted microscope (Nikon U.K. Ltd.) equipped with a Narishige MM0-202N hydraulic three-dimensional micromanipulator (Narishige Inc.). To deplete *BECN1* in germinal vesicle (GV) oocytes, 10 pl (1 μg/μl) of dsRNA was microinjected into the cytoplasm of the oocytes. After injection, oocytes were cultured for 24 h in TCM-199 medium containing 1 mM dbcAMP. Oocytes were then transferred to fresh TCM-199 medium and cultured for 48 h. Control oocytes were microinjected with 10 pl of water.

### Drug Treatment

LY294002 (Sigma) was used as an autophagy-selective inhibitor. To investigate the function of autophagy, LY294002 was added to IVM medium to final concentrations of 1 μM. This concentration of LY294002 was selected on the basis of our experiments on parthenogenetically activated embryos of porcine which can effectively inhibit autophagy.

### Quantitative RT-PCR with SYBR Green

Total RNA was extracted using the Dynabeads mRNA Direct Kit (Dynal Assay) according to the manufacturer’s instructions. First-strand cDNA was synthesized by reverse transcription of mRNA using Oligo (dT) 12–18 nucleotide primers and SuperScript III Reverse Transcriptase (Invitrogen Co.). Real-time PCR (also called quantitative PCR [qPCR]) was performed using a CFX96 Touch Real-Time PCR Detection System (Bio-Rad) in final reaction volumes of 20 μl including SYBR Green, a fluorophore that binds dsDNA (qPCR kit from Finnzymes). The PCR conditions were as follows: 95 °C for 10 min followed by 39 cycles of 95 °C for 30 s, 60 °C for 30 s, and 72 °C for 25 s, and a final extension at 72 °C for 5 min. Finally, gene expression was quantified using the 2^−ΔΔCt^ method and normalized against the mRNA levels of glyceraldehyde 3-phosphate dehydrogenase (*GAPDH*). The primers used to amplify each gene are listed in Table 1.

**Table 1:** List of Primers used in this work.

Gene	Accession No.	Primer sequence (5′-3′)	Product size	Efficiency	R^2^
Gapdh	AF017079	F: GGGCATGAACCATGAGAAGT R: AAGCAGGGATGATGTTCTGG	230	103.3%	0.997
Lc3	NM_001190290	F: CCGAACCTTCGAACAGAGAG R: AGGCTTGGTTAGCATTGAGC	206	102.5%	0.991
Becn1	NM_001044530	F: AGGAGCTGCCGTTGTACTGT R: CACTGCCTCCTGTGTCTTCA	189	98.1%	0.995

F, forward; R, reverse.

### Immunofluorescence analysis

Oocytes or embryos were washed with PBS, fixed in 3.7% paraformaldehyde (w/v) in PBS containing 0.1% PVA, and permeabilized with 1% Triton X-100 (v/v) for 1 h at 37 °C. For detection of 5mC, permeabilized embryos were additionally incubated in 4 N HCl solutions at room temperature for 10 min followed by neutralization in Tris-HCl, pH 8.0, for 10 min. Samples were blocked with 1% BSA (w/v) for 1 h, incubated overnight with the appropriate antibody at 4 °C in a blocking solution, and washed with 1% BSA. The primary antibodies used were rabbit anti-γH2AX (pS139, 1:100; cat. no. 2577; Cell Signaling Technology), rabbit anti-BECN1 (1:100; cat. no. SAB4503706; Proteintech), mouse anti-LC3 (1:100; cat. no. 4937; Proteintech) and mouse anti-5mC antibodies (1:100; cat. no. NA81; Calbiochem). Oocytes or embryos were washed three times with PBS containing 1% BSA and labeled with Alexa Fluor 546 donkey anti-rabbit/Alexa Fluor 488 donkey anti-mouse (Invitrogen) for 1 h at room temperature. Oocytes were then counterstained with 5 μg/ml Hoechst 33342 (bisBenzimide H33342 trihydrochloride; Sigma Life Science) for 15 min, washed three times with PVA-PBS, mounted on a glass slide, and examined using an LSM 710 META confocal laser-scanning microscope (Zeiss). γH2AX foci larger than 0.3 μm^3^ in each nucleus were considered to be sites of DNA double-strand breaks.

### Annexin-V staining of oocytes

For the detection of early-apoptosis, an Annexin-V staining kit was used (cat. no. APOAF; Sigma). Briefly, 30–50 live oocytes were washed twice in PBS and stained for 10 min in the dark with 100 ml of binding buffer containing 10 ml of Annexin-V-FITC according to the manufacturer’s instructions. Fluorescence was measured through using fluorescence microscopy with 450–490 nm (excitation) and 520 nm (emission) filters.

### Western blotting

200 embryos were collected in SDS sample buffer (10 mmol/L, pH 6.8, Tris–Cl, 20 mmol/L DTT, 4% SDS, 0.2% bromophenol blue, and 20% glycerol), respectively, and heated to 100 °C for 5 min. The total proteins were separated by SDS–PAGE with a 5% stacking gel and 12% separating gel at 60 V, 0.5 h and 100 V, 2 h, respectively, and then electrophoretically transferred to nitrocellulose membrane (Bio-Rad Laboratories, Hercules, CA, USA) for 1.5 h, 0.65 mA/cm^2^. Following transfer, blocking in 5% skimmed milk in TBST (TBS containing 0.1% Tween 20) at 4 °C overnight, the membrane was incubated in TBST containing 1:1000 mouse anti-LC3 (cat. no. 4937; Proteintech) or GAPDH antibody(cat. no. 97166; Cell Signaling technology) at 37 °C for 2 h. The membrane was then incubated with horseradish peroxidase-conjugated secondary antibodies (Santa Cruz Biotechnology, Santa Cruz, CA, USA) diluted 1:1000 in TBST at 37 °C for 1 h. Signals were detected using Pierce ECL Western blotting substrate (Thermo Fisher Scientific). To quantify Western blot results, band intensity values were determined using ImageJ software.

### Reactive oxygen species staining

Oocytes were incubated for 15 min in IVC medium containing 10 μM 2′,7′-dichlorodihydrofluorescein diacetate (H_2_DCF-DA) at 37 °C. After incubation, oocytes were washed three times with IVC medium and transferred to PBS drops covered with paraffin oil in polystyrene culture dishes. Fluorescent signals were captured using an epifluorescence microscope (Nikon Corp., Tokyo, Japan). The fluorescence intensity in the control group was arbitrarily set at 1, and the fluorescence intensities in the treatment groups were then measured and expressed as values relative to that of the control group.

### Mitochondrial membrane potential assay

To measure mitochondrial membrane potential (Δφm), blastocysts were washed three times with PBS and incubated in culture medium containing 0.5 μM 5,5′,6,6′-tetrachloro-1,1′,3,3′-tetraethyl-imidacarbocyanine iodide (JC-1) (Invitrogen, Grand Island, NY, USA) at 37 °C in 5% CO_2_ for 30 min. Membrane potential was calculated as the ratio of red florescence, corresponding to strongly activated mitochondria (J-aggregates), to green fluorescence, corresponding to less-strongly activated mitochondria (J-monomers). Fluorescence was visualized with a Zeiss inverted confocal microscope equipped with a 40× oil immersion objective (Zeiss, Jena, Germany). Images were processed with ZEN software (Zen Software, Manchester, UK). The fluorescence intensity in the control group was arbitrarily set to 1, and the relative fluorescence intensity in the treatment groups determined relative to that of controls. Three separate experiments were performed with 10–15 oocytes in each.

### Comet assay

Comet assays were performed using an OxiSelect Comet Assay Kit (cat. no. STA-350; Cell Biolabs). Prior to the assay, OxiSelect comet agarose was melted at 90 °C for 20 min and then cooled at 37 °C for 20 min. Approximately 75 μl of agarose was then dropped onto an OxiSelect 3-well comet slide, after which approximately 20 zona-free embryos were transferred to agarose drops and chilled at 4 °C for 20 min. Samples were then lysed in lysis buffer (250 Mm NaCl, 20% EDTA solution, 10% DMSO, 10% 10× kit lysis solution, pH 10.0) at 4 °C for 2 h. Next, slides were carefully transferred to a chilled alkaline solution (300 mM NaOH, 1 mM EDTA) and immersed for 30 min at 4 °C. Subsequently, slides were transferred to a horizontal electrophoresis chamber filled with cold TAE buffer and electrophoresed for 20 min at 25 V, after which the slides were stained with 1× Vista Green DNA Dye for 15 min and then examined using a fluorescent microscope with a FITC filter. All steps after agarose treatment were conducted in the dark to prevent additional DNA damage. Comet tail lengths were measured in individual oocytes using CASP (ver. 1.2.3beta2; Zbigniew Koza).

### Fluorescence Intensity Analysis

ImageJ software (v.1.47) was used to define regions of interest (ROI), and the average fluorescence intensity per unit area within each of the ROI determined. Independent measurements of the cell nucleus or cytoplasm were made using ROI of identical size. The average values of all measurements were used to compare the final average intensities between control and treated oocytes.

### Statistical analysis

The general linear models (GLM) procedure in the Statistical Analysis System (SAS User’s Guide, 1985, Statistical Analysis System Inc., Cary, NC) was used to analyze the data from all the experiments. Significant differences were determined using Tukey’s multiple range test and P < 0.05 was considered significant.

## Results

### Expression and Subcellular Localization of Autophagy during Porcine Oocyte Maturation

**Figure 1:**
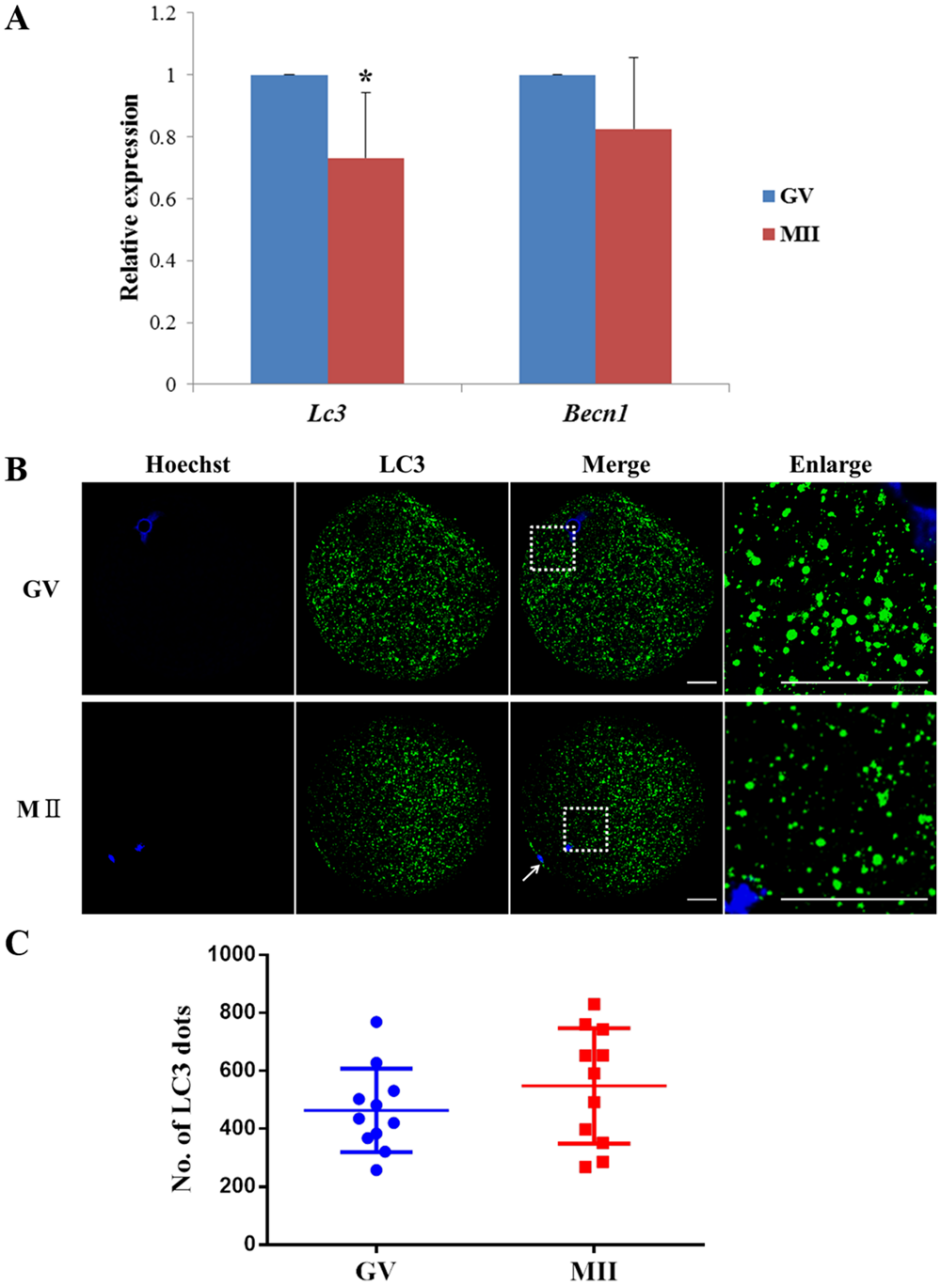
Localization and expression of autophagy related gene in GV (0 h) and MII (44 h) stage oocytes. (**A**) Relative mRNA expression levels of *LC3* and *BECN1* at the GV and MII stage oocytes analyzed by qRT PCR. mRNA expression at the GV stage was arbitrarily set as onefold. Fold differences in the mRNA expression from equivalent numbers of GV and MII stage embryos are shown after normalisation against the internal standard *GAPDH*. Data are presented as the mean ± SEM. ^*^P < 0.05. (**B**) Oocytes at the GV and MII stage were immunolabeled with anti-LC3 antibody and Hoechst33342 to visualize the localization of LC3 in porcine oocytes. The MII polar body is indicated by a white arrow. In the enlarged panel (indicated by the white box) dots represent the localization of LC3. Scale bar = 20 μm. (**C**) Quantification of LC3 dots in oocytes. Each value represents the mean ± SEM.

To investigate the expression pattern of autophagy during porcine oocyte maturation *in vitro*, we firstly examined the quantity of Lc3 and Becn1 mRNA in germinal vesicle (GV) formation and metaphase II (MII) stage porcine oocytes. Both GV and MII oocytes showed similar pattern of Lc3 and Becn1 mRNA expression, which was higher at the GV stage and slightly decreased in the MII stage (Fig. 1A).

Next, we examined the subcellular localization of LC3 during oocyte maturation by immunofluorescent staining. Conversion of cytosolic LC3 (LC3-I) to membrane bound phosphatidylethanolamine (PE)-conjugated LC3 (LC3-II) occurs during autophagy induction, and the amount of LC3-II correlates with the number of autophagosomes. Our results indicated that numerous of autophagosomes (LC3-II punta) appeared in oocytes both of the GV and MII stages (Fig. 1B,C).

### Autophagy is essential for first polar body extrusion

**Figure 2:**
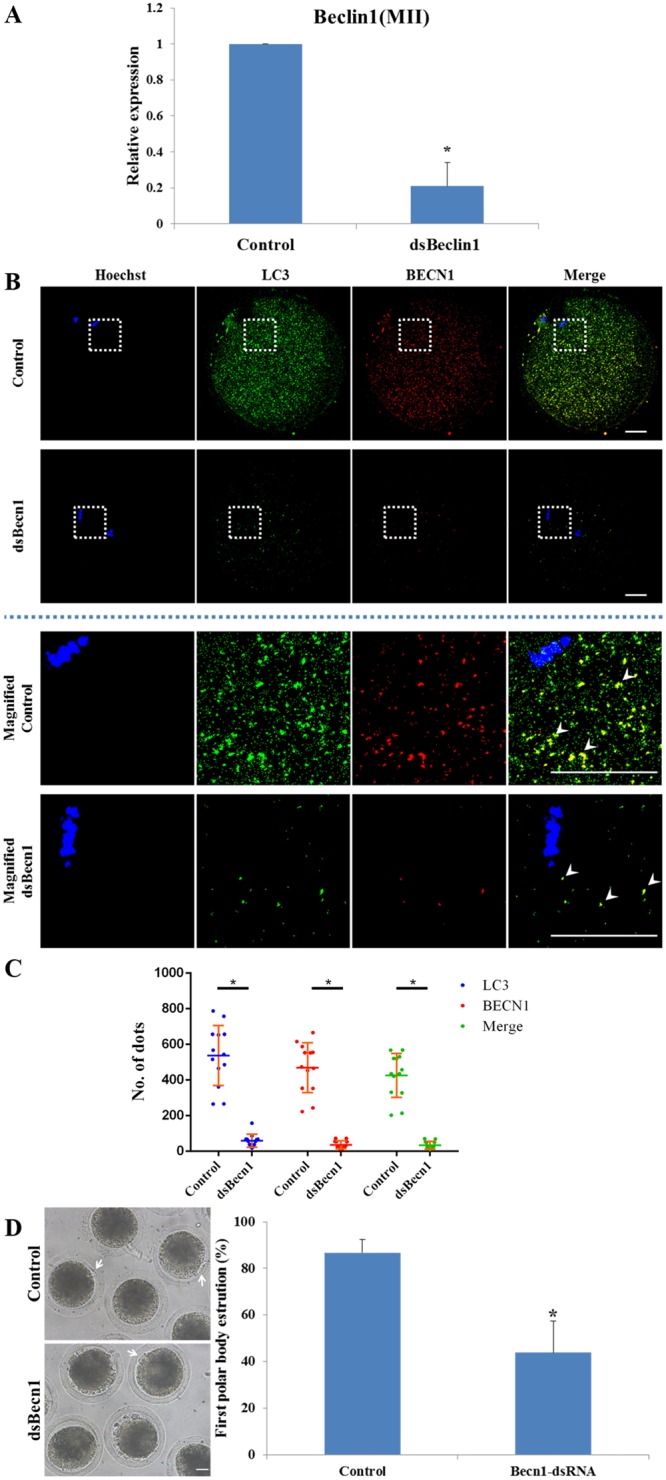
*BECN1* dsRNA inhibits extrusion of the first polar body. (**A**–**C**) Knockdown of endogenous *BECN1* mRNA and protein expression after *BECN1* dsRNA injection was verified by qRT-PCR (**A**) and immunofluorescent staining (**B**,**C**). BECN1 mRNA and protein expression were significantly decreased after dsRNA injection. In the enlarged panel (indicated by the white box) dots represent the localization of LC3 labelled protein, BECN1 labelled protein. Note that the colocalisation of LC3 and BECN1. Scale bar = 20 μm. Each value represents the mean ± SEM. ^*^P < 0.01. (**D**) Effect of BECN1 knockdown on the rate of oocyte polar body extrusion after 44 h of *in vitro* culture. The MII polar body is indicated by a white arrow. Scale bar = 20 μm. Each value represents the mean ± SEM. ^*^P < 0.05.

**Figure 3:**
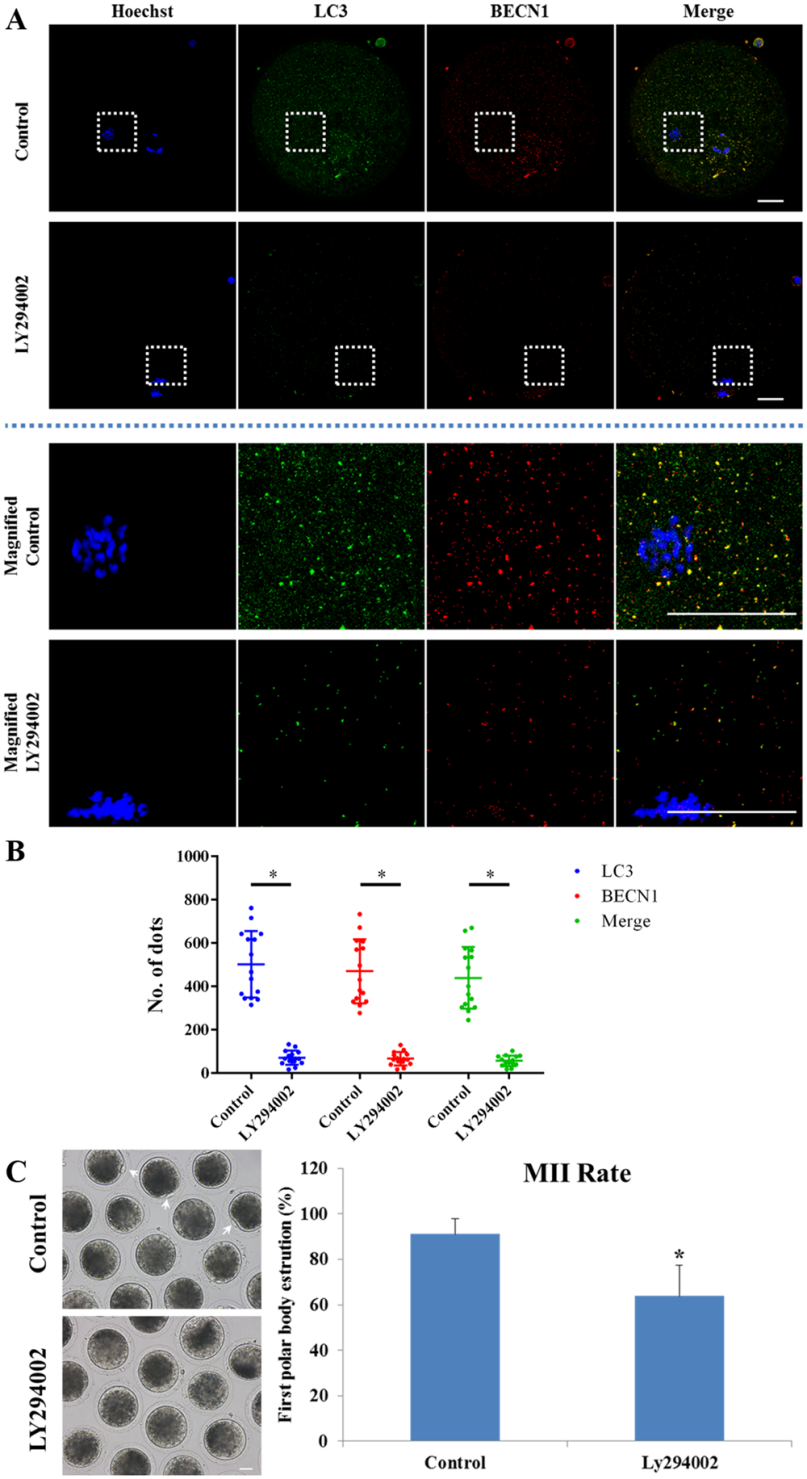
Effects of autophagy inhibition on porcine oocyte maturation. (**A**,**B**) LC3 and BECN1 protein expression after 1 μM LY294002 treatment was determined by immunofluorescent staining. BECN1 and LC3 protein expression were significantly decreased after LY294002 treatment. In the enlarged panel (indicated by the white box) dots represent the localization of LC3 labelled protein, BECN1 labelled protein. Scale bar = 20 μm. Each value represents the mean ± SEM. *P < 0.01. (**C**) Effect of LY294002 treatment on the rate of oocyte polar body extrusion after 44 h of *in vitro* culture. The MII polar body is indicated by a white arrow. Scale bar = 20 μm. Each value represents the mean ± SEM. ^*^P < 0.05.

**Figure 4:**
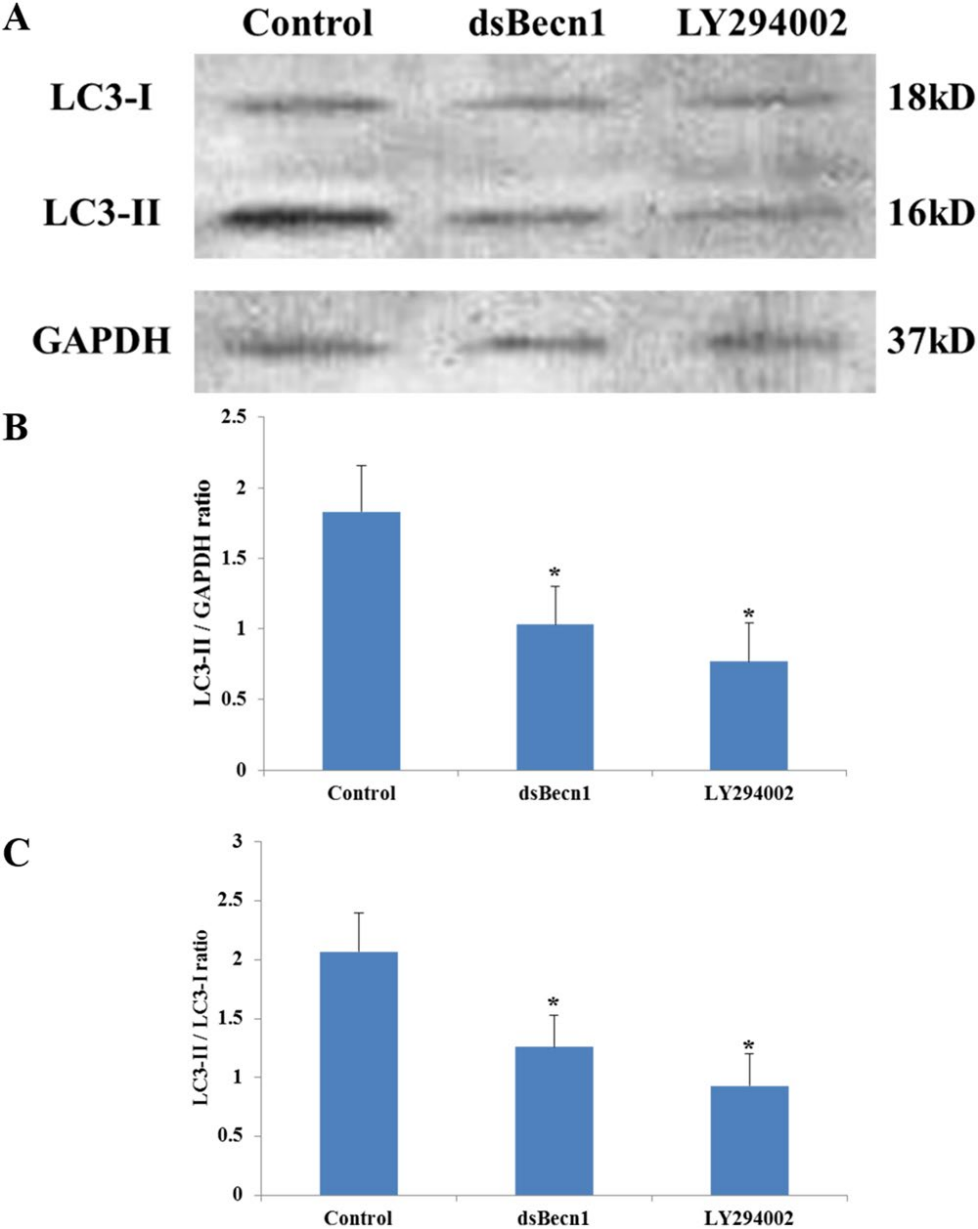
Western blotting assay for conversion of LC3-I (cytosolic) to LC3-II (autophagosome bound). (**A**) LC3- I (lane 1), LC3-II (lane 2) and GAPDH (lane 3) in untreated MII (Control), dsBECN1-injected (dsBECN1) and LY294002 treated (LY294002) oocytes. Graphs (**B**,**C**) show LC3-II quantification by western blotting in oocytes in different groups. While B shows LC3-II contents (LC3-II/GAPDH ratio), (**C**) shows the LC3-II/ LC3- I ratio. ^*^P < 0.05.

To assess whether autophagy is required during porcine meiotic maturation, we inhibited autophagic activity by knocking down the expression of *BECN1*, using dsRNA or the autophagy selective inhibitor, LY294002. The level of *BECN1* mRNA was significantly decreased after dsRNA injection (Fig. 2A). Immunofluorescent staining method was used for analysis the number of autophagosomes. The number of LC3 and BECN1 puncta formation was very low both in Becn1 knockdown and LY294002 treatment group compared with the control group (Figs 2B,C, 3A,B). Additionally, induction of autophagy was also confirmed by LC3 conversion. Western blot analysis showed that the amount of LC3-II was also less than control group in the Becn1 knockdown or LY294002 treatment oocytes (Fig. 4). As shown in Fig. 2D, the majority (83.68%) of control denuded oocytes underwent MII at 44 h of culture, compared with only 47.67% of those with *BECN1* expression knocked down. After 44 h of treatment with LY294002, the rate of MII was reduced (62.31%), compared with that of the control group (88.39%) (Fig. 3C).

### Autophagic inhibition triggers DNA damage and early apoptosis in porcine oocytes

**Figure 5:**
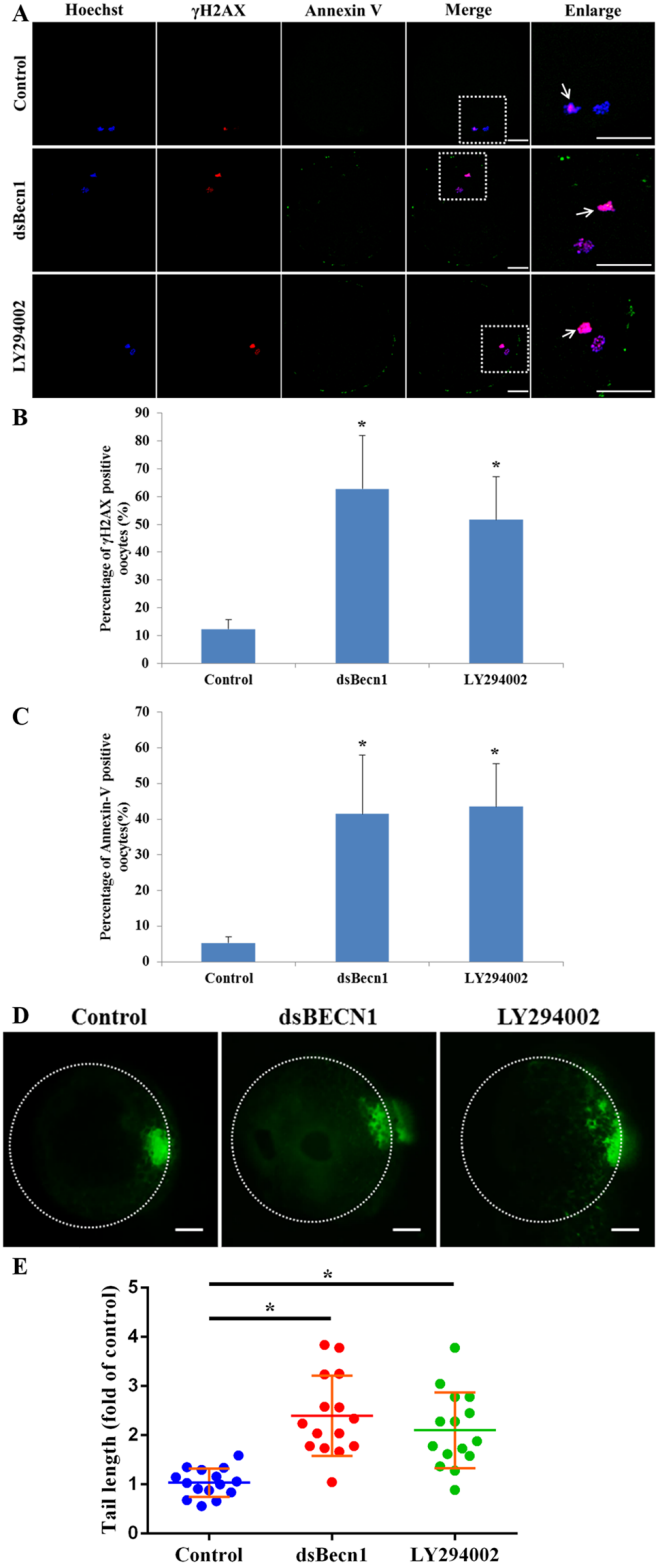
DNA damage and apoptosis of oocytes after dsBECN1 injection or autophagy inhibitor treatment. (**A**) Localization of γH2A.X in the nuclei of oocytes and early apoptosis in the membrane of oocytes by performing annexin-V staining. The percentage of γH2A.X positive and annexin-V positive oocytes significantly increased after injection of dsBECN1 or LY294002 treatment. Blue, DNA; red, γH2A.X; green, Annexin-V. The MII polar body is indicated by a white arrow. Scale bar = 20 μm. (**B**) Quantification of γH2A.X positive oocytes. Each value represents the mean ± SEM. *P < 0.01. (**C**) The percentage of annexin-V-positive oocytes. Each value represents the mean ± SEM. ^*^P < 0.01. (**D**) DNA damage in oocytes was assessed by performing the comet assay. Control oocytes showed slight DNA damage, whereas dsBECN1-injected or LY294002-treated oocytes showed notable DNA damage. Scale bar = 20 μm. (**E**) Fold changes in tail moment and length in oocytes. Each value represents the mean ± SEM. ^*^P < 0.01.

We found that phosphorylated H2AX (γH2A.X) was abundant in the nuclei of dsBECN1-injected and LY294002-treated MII oocytes, whereas it was almost absent in the nuclei of controls. Moreover, levels of γH2A.X were markedly increased in dsBECN1-injected or LY294002-treated oocytes compared with those in control oocytes (P < 0.01; Fig. 5A,B). These data suggest that inhibition of autophagy induces a DNA damage response in oocytes. Results of DNA comet assay further confirmed the occurrence of DNA damage in autophagy inhibited oocytes (p < 0.05; Fig. 5D,E).

We next performed annexin V-FITC staining to explore whether inhibition of autophagy induced early apoptosis in MII oocytes. The results showed that the percentage of oocytes emitting green fluorescence in cell membrane was significantly higher among dsBECN1-injected or LY294002-treated oocytes than among control oocytes (P < 0.01; Fig. 5A,C).

### Autophagy inhibition affects mitochondrial function in porcine oocytes

**Figure 6:**
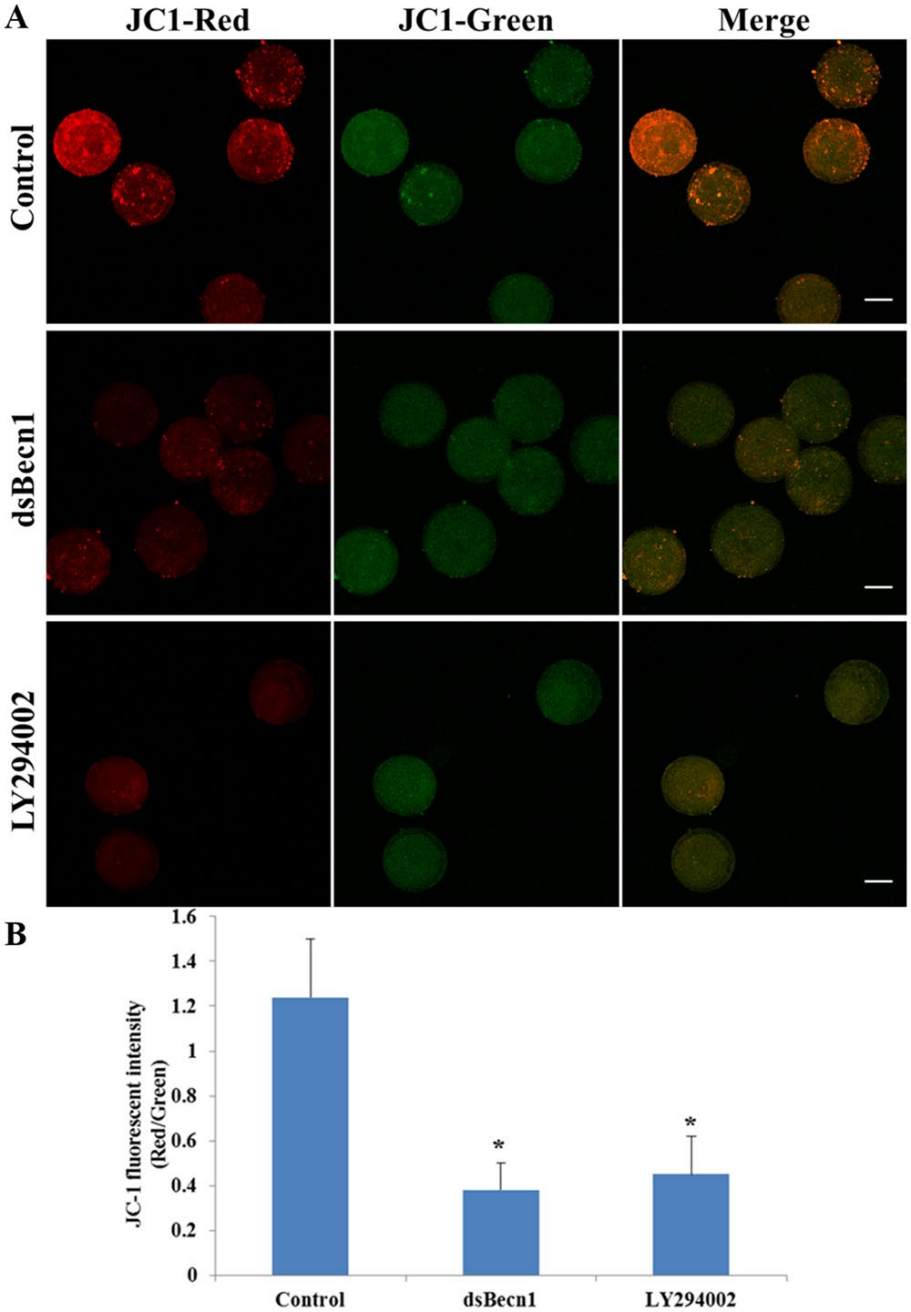
Mitochondrial potential in oocytes after dsBECN1 injection or autophagy inhibitor treatment. (**A**) JC-1 staining of dsBECN1-injected or LY294002-treated oocytes. ΔΨm was significantly lower in dsBECN1-injected or LY294002-treated oocytes than in control oocytes. Scale bar = 50 μm. (**B**) Fluorescence intensity of JC-1 in oocytes. Each value represents the mean ± SEM. ^*^P < 0.01.

The mitochondria of mammalian cells are key to the production of cellular energy. Therefore, to determine whether autophagic inhibition can affect mitochondrial function, we evaluated mitochondrial ΔΨm in porcine MII oocytes. Representative images of mitochondrial ΔΨm are presented in Fig. 6A. Average ΔΨm values were significantly decreased in dsBECN1-injected or LY294002-treated oocytes compared with those in controls (P < 0.01) (Fig. 6B).

### Autophagy inhibition induces ROS formation in porcine oocytes

**Figure 7:**
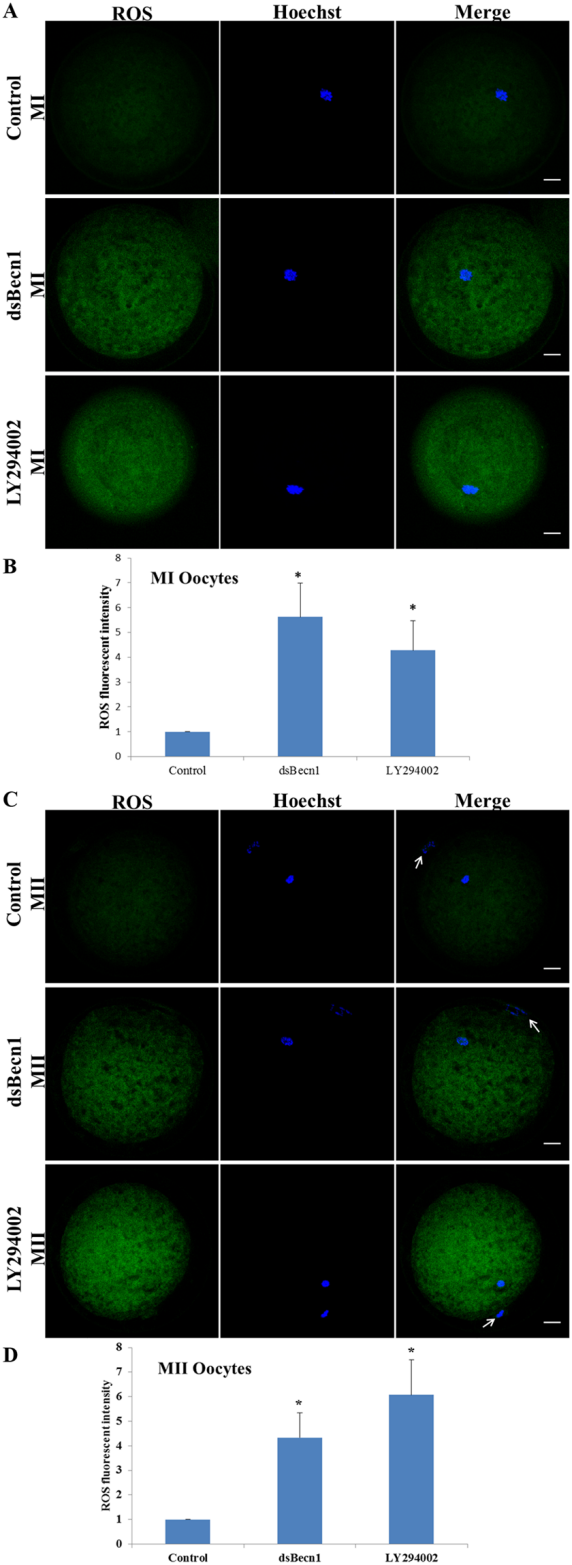
ROS content in oocytes after dsBECN1 injection or autophagy inhibitor treatment. (**A**) ROS in MI oocytes stained with DCDHF (green). Scale bar = 20 μm. (**B**) Relative fluorescence intensity of ROS. (**C**) ROS in MII oocytes stained with DCDHF (green). The MII polar body is indicated by a white arrow. Scale bar = 20 μm. (**D**) Relative fluorescence intensity of ROS. Control data values were arbitrarily set at 1. Values represent mean ± SEM from at least three separate experiments. ^*^P < 0.01.

To investigate why autophagic inhibition led to decreased mitochondrial membrane potential, ROS content in MI and MII stage oocytes was determined. ROS content was relatively elevated in dsBECN1-injected or LY294002-treated MI and MII stage oocytes (Fig. 7A,C), with fluorescence intensities in the dsBECN1-injected or LY294002-treated groups significantly higher than those of controls (P < 0.01) (Fig. 7B,D).

### Autophagy inhibition during oocyte maturation affects the capacity of porcine oocytes for further development

**Figure 8:**
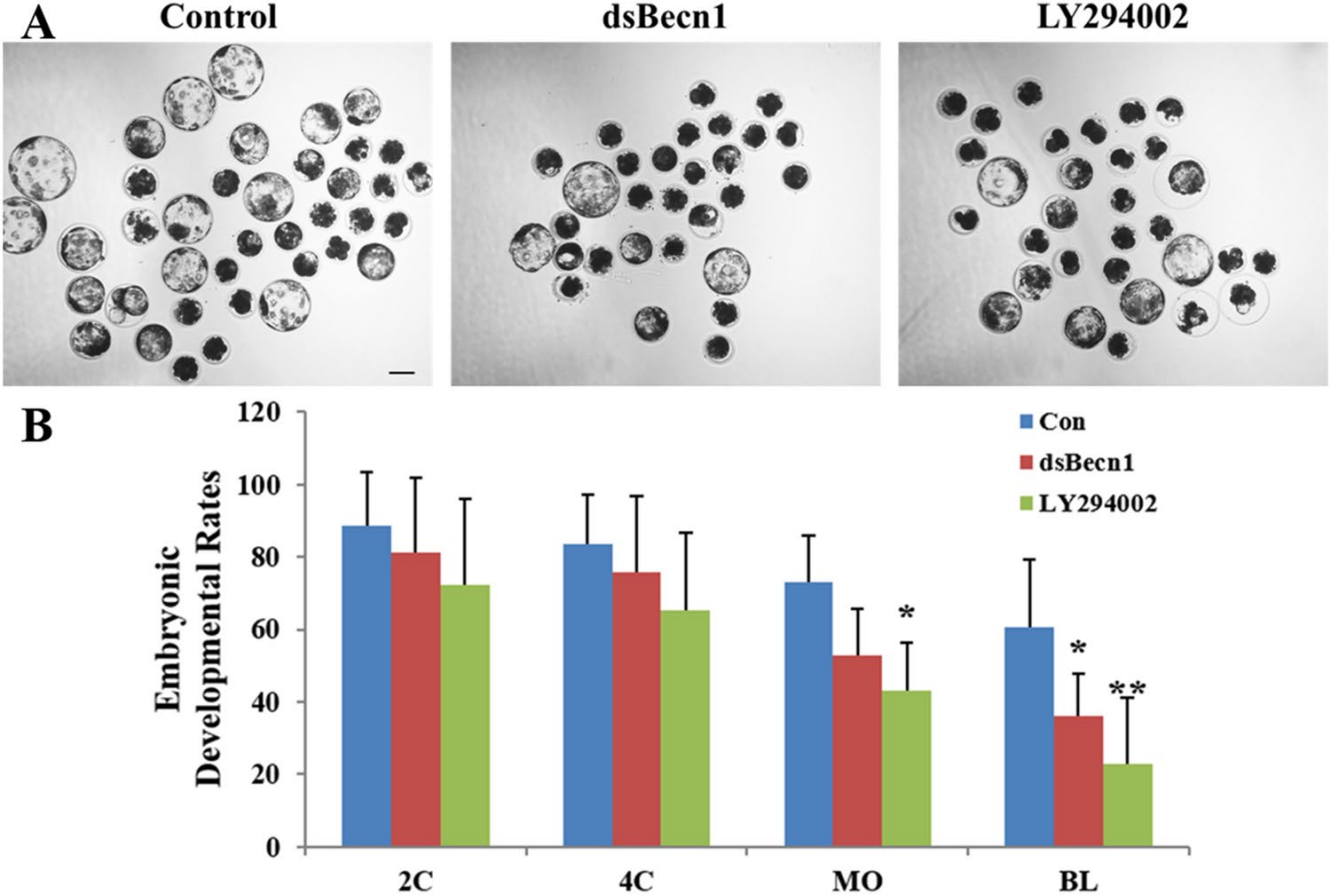
Preimplantation development after dsBECN1 injection or autophagy inhibitor treatment during IVM. (**A**) dsBECN1-injection or LY294002 treatment during oocyte maturation decreased the developmental potency of oocytes after parthenogenetic activation. Scale bar = 100 μm. (**B**) Embryonic development rates of control and dsBECN1-injected or LY294002-treated oocytes compared with control oocytes. ^*^P < 0.05, ^**^P < 0.01.

To examine the effects of autophagy inhibition on the capacity of porcine oocytes for further development, zygotes produced by parthenogenetic activation of MII oocytes were monitored for their developmental potential (Fig. 8). In control embryos, the developmental rate began to decline after the first cleavage, with 60.4% of activated embryos successfully developing to the blastocyst stage 160 h post-activation (Fig. 8A,B). Strikingly, embryos injected with dsBECN1 or treated with LY294002 developed to the blastocyst stage with low efficiency (36.1% and 22.8%, respectively; Fig. 8A,B).

## Discussion

A landmark study demonstrated a critical role for autophagy in early mammalian preimplantation development, at the stage of transition from the maternal to the zygotic genetic program in mouse^15^; however, autophagy does not occur during meiosis in mice and rats^15,17^. Our results suggest that autophagy is triggered during porcine oocyte maturation. This result is in accordance with a previous report that autophagosomes are present during the *in vitro* maturation of porcine oocytes^18^. As autophagy is a primary response to cellular stress, and an attempt to survive unfavorable conditions, such as starvation, heat, or hypoxia, it is possible that it is normally repressed, but activated in response to stress conditions during oocyte meiosis, including porcine oocytes during 44 h of *in vitro* maturation^18^. Consistent with this hypothesis, autophagy activation has been reported in vitrified-warmed oocytes^19^, and induction of autophagy by treatment with rapamycin (an mTOR inhibitor) improves nuclear and cytoplasmic maturation and preimplantation development of porcine oocytes and embryos^20^.

Oocyte maturation is complex and errors during this process can prevent fertilization and block embryo development^21^. To produce good quality porcine embryos, it is important to prepare MII oocytes with high developmental competence. Mitochondria play a pivotal role during oocyte maturation, and mitochondrial dysfunction has been implicated in the induction of developmental retardation and arrest of embryos^22^. Inner mitochondrial transmembrane potential (ΔΨm) is commonly used as an indicator of mitochondrial function and the viability of oocytes^23^. ΔΨm reflects the activity of hydrogen ion pumps within the membrane-bound electron transport chain, which are the driving force of ATP production. Damaged mitochondria are particularly prone to activating the apoptotic program^24^. Our results demonstrate that inhibition of autophagy disrupts oocyte ΔΨm. Autophagy plays an important role in the regulation of mitochondrial function; mitochondrial membrane depolarization precedes the induction of autophagy and autophagy is induced to protect against different types of mitochondrial stress by inhibition of depolarization^25^.

ROS disrupt mitochondrial function and play a significant role in oocyte maturation^26^. Moreover, porcine oocytes have a higher lipid content than those of other species and are highly sensitive to ROS-induced damage^27^. Our results show that decreased autophagy induces excessive ROS generation in porcine oocytes. A previous study showed that sustained exposure to ROS induces mitochondrial damage^28^ and prevents the development of embryos cultured *in vitro*^29^. It is widely accepted that autophagy is crucial for the removal of damaged mitochondria. ROS generated by damaged mitochondria may induce mitophagy, which in turn eliminates the damaged organelles, leading to decreased levels of ROS. Autophagic regulation is associated with apoptosis induction and involves DNA damage. Recent studies indicate that autophagy-induced apoptosis is regulated by a ROS-associated mitochondrial pathway^30^. These findings prompted us to investigate the effects of autophagy on DNA damage and apoptosis in porcine oocytes. Our results show that inhibition of autophagy induces the accumulation of DNA damage in porcine oocytes, as determined by analysis of γH2A.X levels, a common biomarker of cellular responses used for monitoring DNA damage and repair, and comet assays^31–33^. Thus, inhibition of autophagy may arrest porcine oocyte maturation by increasing the accumulation of DNA damage. Apoptosis is a complex process that may be induced by DNA damage in oocytes^34^. Our results suggest that autophagy inhibition increases the incidence of early apoptosis. As both DNA damage and early apoptosis affect oocyte meiotic maturation^35,36^, these are potential mechanisms through which autophagic inhibition affects porcine oocyte meiotic maturation and blocks their further development.

Both nuclear maturation and cytoplasmic maturation are important for development potential of oocytes after fertilization or activation. Compared to nuclear maturation, cytoplasmic maturation is a complicated process that is regulated by mechanisms that are not well known. Taking consideration of the fact that the potential of nuclear matured oocytes to develop to blastocysts in autophagic inhibited groups was significantly damaged when compared to the control group, we suggest that the low developmental ability of the matured oocytes in treatment groups are probably due to the insufficient cytoplasmic maturation.

In conclusion, we demonstrate for the first time the involvement of autophagy in the maturation of porcine oocytes. Our results show that autophagic inhibition during porcine oocyte maturation exerts detrimental effects on polar body extrusion. Inhibition of autophagy not only induced DNA damage and apoptosis in porcine *in vitro* matured oocytes, but also disrupted oocyte mitochondrial membrane potential, thus affecting the embryonic developmental potential of porcine oocytes. Our findings suggest that autophagy is important for the *in vitro* maturation of oocytes and further development of porcine embryos.
